# Effects of Dietary Nano-Composite of Copper and Carbon on Antioxidant Capacity, Immunity, and Cecal Microbiota of Weaned Ira White Rabbits

**DOI:** 10.3390/ani15020184

**Published:** 2025-01-11

**Authors:** Ying-Huan Zhou, Xiao-Ping Liu, Xiao-Ming Gu, Hai-Xuan Lv, Yun Yang, Zai-Xing Cai, Bin Di, Chang-Kang Wang, Yu-Yun Gao, Ling Jin

**Affiliations:** 1 College of Animal Sciences, Fujian Agriculture and Forestry University, Fuzhou 350002, China; yh980125@163.com (Y.-H.Z.); xiaopingliu2412@163.com (X.-P.L.); 15137655073@163.com (X.-M.G.); haixvanlv2024@163.com (H.-X.L.); 15507386602@163.com (Y.Y.); czaixing0410@163.com (Z.-X.C.); 18362272307@163.com (B.D.); wangchangkangcn@163.com (C.-K.W.); 2 China National Engineering Research Center of JUNCAO Technology, Fujian Agriculture and Forestry University, Fuzhou 350002, China

**Keywords:** NCCC, weaning stress, liver antioxidant, jejunal immunity, cecum microorganisms, Ira white rabbit

## Abstract

This experiment investigated the effects of dietary supplementation with nano-composites of copper and carbon (NCCC) on antioxidants, immune functions, and the cecum microbiota of weaned Ira white rabbits. A total of 240 weaned 35-day-old Ira white rabbits were randomly allocated to five dietary treatments (*n* = 6 per treatment, each replicate consisted of eight rabbits) that included the control group (CON) with a basal diet, the SAL group with 60 mg/kg salinomycin (SAL) in addition to the basal diet, and the NCCC I, II, III groups, which were supplemented with 50, 100, and 200 mg/kg NCCC, respectively, in addition to the basal diet. The test lasted for 28 d. The results showed that dietary NCCC supplementation increased the liver Cu/Zn-SOD content and up-regulated the gene expression of Cu/Zn-SOD (*p* < 0.05), while also reducing the content of MDA in the liver and enhancing the antioxidant capacity of Ira white rabbits. Moreover, the NCCC diet supplementation reduced the content of IL-6 and down-regulated the relative expression of *IL-6* and *IL-1β* genes in the jejunum of Ira white rabbits (*p* < 0.05). In addition, the metagenomic analysis of 16 S rRNA showed significant differences in the cecal microbial structure of weaned Ira white rabbits in the NCCC III group compared with the CON, NCCC I, and NCCC II groups (*p* < 0.05). Firmicutes and Bacteroidetes were the dominant phyla of cecal microorganisms in weaned Ira rabbits in the NCCC diet groups. The dominant genera included unidentified *Eubacteriaceae*, unclassified *Lachnospiraceae*, *Christensenellaceae*, and *Ruminococcus*. Furthermore, the relative abundance of *Ruminococcus* in the NCCC I and II groups was lower than that in the CON group in the cecum of Ira white rabbits (*p* < 0.05). In summary, our results showed that diet supplementation with NCCC could enhance the antioxidant capacity in the liver, alleviate intestinal inflammation, and regulate the structure of intestinal flora, improving the health of Ira white rabbits.

## Simple Summary

Nano-composites of copper and carbon (NCCC) are a novel nanoparticle material. They are synthesized using plant fibers as templates and copper ions as the copper source via a carbonization reduction method. NCCC, as a novel feed additive, possesses attributes such as low dosage requirements, stability, and the potential to mitigate environmental pollution. In the present study, dietary supplementation with NCCC increased the liver Cu/Zn-SOD levels, reduced MDA content, and enhanced antioxidant capacity of Ira white rabbits. Additionally, it decreased the levels of pro-inflammatory cytokines *IL-6* and *IL-1β* in the jejunum. Furthermore, metagenomic analysis indicated notable changes in cecal microbial composition, improving the flora structure. In summary, NCCC supplementation improves liver antioxidant capacity, mitigates intestinal inflammation, and alters gut microbiota, thereby promoting the health of Ira white rabbits.

## 1. Introduction

Rabbits not only can provide emotional companionship as companion animals due to their docile nature, but due to their unique biological characteristics, such as rapid reproduction, short life cycle and sensitivity to a wide range of pathogens, they are widely used in scientific research, production, teaching and other scientific experiments [1,2,3]. In the field of meat production, on the other hand, the rabbit’s highly efficient growth rate and high-quality meat characteristics have made it an important part of the agricultural economy [4,5]. According to the FAOSTAT database of the Food and Agriculture Organization (FAO), between 2000 and 2022, rabbit meat reached about 13 million tons of China’s total meat production, ranking first in the world and reflecting the important contribution of the rabbit to the global agricultural economy [6]. In contemporary meat rabbit farming, weaning is typically performed at approximately 35 days of age, with a variation of ±2 days. However, the weaning rabbits are highly susceptible to weaning stress syndrome due to changes in their nutritional sources, imperfect development of their immune function, and changes in the external environment, resulting in diarrhea and even death [7,8,9]. Diarrhea is a common ailment in young rabbits and may lead to secondary infections, resulting in intestinal microbial imbalances, reduced immunity, and increased mortality rates [10], which further hinder the growth of rabbit farming. Antibiotic growth promoters are used as the standard prevention strategy on commercial farms [11,12]. However, this routine use of antibiotics is decreasing, due to legal restrictions in some countries and the growing concern of consumers about the use of antibiotics in animal production, in addition to public health and animal health problems, such as the development of antimicrobial resistance and the scarcity of alternative treatments [13,14,15]. Therefore, it is necessary to find feed additives that can replace antibiotics to regulate the stress response of weaned rabbits.

Novel substitution strategies such as Antimicrobial Peptides (AMP), plant-derived probiotics (e.g., essential oils and plant extracts), and nanotechnology (e.g., nanoparticles) are emerging as hot areas of antibiotic substitution research [16,17,18], which not only present effective solutions to the problem of antibiotic resistance, but also provide new ways to enhance animal health and productivity. Among them, the application of nanotechnology allows metal-containing nanoparticles to achieve higher bioavailability and absorption efficiency at lower doses [19]. This provides an effective way to solve the problem of animal nutritional deficiencies, and increases the content of trace elements in animal products, improving the product quality [20,21]. Therefore, metal-containing nanoparticles as feed additives may have the potential to optimize the nutritional structure and replace antibiotics, making them an efficient and environmentally friendly option. As an essential micronutrient, copper (Cu) plays a crucial role in the formation of key metabolic enzymes, nutrient absorption, and cellular energy metabolism [22,23,24]. The absorption of dietary Cu occurs mainly at the proximal part of the small intestine [25]. Cu is trafficked to special Cu-containing enzymes after being mediated by Cu transport protein 1 in the intestinal epithelium, and then binds to soluble chaperones, such as albumin, transmembrane proteins, and histidine, to perform its function [26,27]. Dietary supplementation with Cu can act as a growth promoter, immunostimulant, antioxidant, and antimicrobial agent for livestock and poultry [28,29]. However, Cu is toxic at high levels, which leads to Fenton-type redox reactions, resulting in oxidative cell damage and cell death [25]. Copper nanoparticles have several advantages over inorganic and organic forms such as a smaller size, larger contact area, and higher bioavailability, which reduce physiological toxicity and environmental contamination due to copper overloading [30], making dietary supplementation with low-dose Cu possible in livestock and poultry production. It has been reported that dietary supplementation with 100 mg/kg of copper-containing chitosan nanoparticles increased the immune function and regulated the structure of microbial communities to promote the growth performance of broilers. [31]. Similarly, dietary 50 mg/kg or 75 mg/kg of copper nanoparticles improved the growth performance, increased the oxidative dismutase activity, and regulated the structure of intestinal flora in New Zealand White rabbits [32].

Nano-composites of copper and carbon (NCCC) are a novel nanoparticle material synthesized through a carbonization reduction method that utilizes plant fibers as templates and copper ions as the source of copper. This “green” synthesis is sustainable, reliable, and environmentally friendly [33]. In addition, NCCC has broad-spectrum antimicrobial and antioxidant capacity which is based on disrupting the physiological functions related to microbial redox balance [34]. Therefore, the purpose of this study was to investigate the effects of dietary supplementation with NCCC on antioxidant and immune functions, as well as the microbiota, of Ira rabbits under weaning stress and to provide a scientific basis for the development and application of NCCC as a replacement antioxidant product and feed additive.

## 2. Materials and Methods

### 2.1. Experimental Design, Animals, and Diets

In order to ensure the accuracy and reliability of the experimental data, The experiment was carried out in Fujian Chunlong Agriculture and Animal Husbandry Technology Co., Ltd. (Fuzhou, China), which has the Quality Management System [1]. Male rabbits were chosen for the experiment due to their physiological characteristics (more regular and stable growth performance and physiological indicators) [35], reproductive factors (avoiding accidental reproduction, simplifying the process of breeding management) and behavioral research (simple behavioral patterns and reduced interference of aggression), as well as other aspects, all of which make male rabbits easier to study and reduce interference. A total of 240 male Ira white rabbits, weaned at the age of 35 days, provided by Fujian Chun Long Agriculture and Animal Husbandry Technology Co., Ltd., were randomly assigned to five treatment groups, with six replicates of eight rabbits. The control group was fed a basal diet, the antibiotic group (SAL) was supplemented with 60 mg/kg salinomycin in addition to the basal diet, and the NCCC I, II, III groups were supplemented with 50, 100, and 200 mg/kg NCCC, respectively, in addition to the basal diet. It should be mentioned that, in order to meet the nutritional needs of meat rabbits, the basal diet contained 0.1 mg/kg copper. This trial started on 14 August 2021, ended on 10 September 2021 and lasted for 28 days. The experimental rabbit cages were 180 cm long, 50 cm wide and 150 cm high, with 8 rabbits in each cage, totaling 30 cages. The basal diet is granular (Fujian Jin Hua Long Feed Co., Ltd., Fuzhou, China), and its composition and nutrient levels are shown in Table 1. NCCC, composed of 12% copper and 88% carbon, was provided by Xiamen C&D Tong Shang Co., Ltd. NCCC has a copper core (diameter: 20 to 50 nm) surrounded by a carbon shell (diameter: about 15 μm), and its production process comprises loading copper ions into plant fibers and then carbonizing the fibers [17]. The indoor temperature was controlled at 24 °C and humidity at 65%. The rabbit hutches were provided with 16 h of light and 8 h of darkness cyclic light conditions daily. All rabbits had ad libitum access to feed and water. All the experimental procedures applied in this study were reviewed and approved by the Committee of Animal Experiments of Fujian Agriculture and Forestry University (Fuzhou, Fujian, China, approval ID 202003013).

### 2.2. Sample Collection

At 28 d, 1 weaned rabbit was selected from each replicate in each group for slaughter sampling, totaling 30 rabbits. Euthanasia was performed using a marginal ear intravenous injection of sodium pentobarbital, which was performed by technicians who were experienced laboratory technicians to ensure the accuracy of the operation and minimal pain to the animal. The abdominal cavity of the rabbits was dissected using sterile dissecting scissors along with a scalpel. The liver tissues were harvested, coded, and snap-frozen in a liquid nitrogen tank, and then maintained at −80 °C for further analysis. The content of the cecum was collected in a cryopreservation tube and kept at −80 °C for 16S rRNA analysis. The jejunum and cecal were opened longitudinally with sterile forceps and gently rinsed with 4 °C phosphate-buffered saline (PBS). A sample of the jejunal and cecal mucosa was gently scraped with a sterile slide, which was then placed in a cryopreservation tube and stored at −80 °C for further analysis.

### 2.3. Liver Antioxidant Indicators

A 0.1 g sample of liver tissue was accurately weighed into a grinding tube and 10% tissue homogenate was prepared by adding a 9-times volume of saline (ratio of liver weight (g):volume (mL) = 1:9) and performing mechanical homogenization (70 Hz, 15 s, 3 times). The liver tissue homogenate was centrifuged for 20 min using a low-speed cryogenic centrifuge (Eppendorf AG, Hamburg, Germany) at 587× *g* to obtain the supernatant. The liver malondialdehyde (MDA, ml094962), ceruloplasmin (CER, ml095306), copper/zinc superoxide dismutase (Cu/Zn-SOD, ml036817), and glutathione peroxidase (GSH-Px, ml095262) were measured in strict accordance with the instructions of the kit (Enzymatic Biotechnology Co., Ltd, Shanghai, China). Absorbance was measured at 450 nm by the iMark™ Microplate Absorbance Meter (Bio-Rad, Hercules, CA, USA).

### 2.4. Determination of Jejunal Mucosal Immune Indicators

A 0.1 g mucosal sample was weighed accurately. Then, homogenization medium was added at 9 times the volume of the sample and the mixture was mechanically homogenized (70 Hz, 15 s, 3 times) to prepare 10% mucosal tissue homogenate. The homogenate was centrifuged at 1000× *g* for 10 min to collect the supernatant. The concentration of IL-6, IL-10, and IL-1β were measured in strict accordance with the instructions of the kit (ml027844, ml027828, and ml027836, respectively; Shanghai EnzymeLinked Biotechnology Co., Ltd., Shanghai, China). Absorbance was measured at 450 nm by the iMark™ Microplate Absorbance Meter (Bio-Rad, Hercules, CA, USA).

### 2.5. qRT-PCR

Total RNA was isolated from liver tissue and jejunum mucosa using the Trans-Zol UP Plus RNA Extraction Kit (Beijing QuanShijin Biotechnology Co., Ltd., Beijing, China). The RNA concentration and purity were assessed using Nanodrop 2000 (Thermo Fisher Scientific Corporation, Wilmington, NC, USA). Subsequently, total RNA was reverse transcribed to cDNA with the PrimeScript RT kit (Promega Biotechnology Co., Ltd., Beijing, China). Finally, quantitative real-time PCR (qRT-PCR) was used to determine the relative gene expression levels under a fluorescence quantitative PCR instrument (Bio-Rad Laboratories Co., Ltd, Shanghai, China), using PerfectStart^®^ Green qPCR SuperMix kit (Promag Beijing Biotechnology Co., Ltd., Beijing, China). qRT-PCR was carried out on the Go Taq^®^ qPCR Master Mix, and the amplification procedure was as follows: initial denaturation at 95 °C for 5 min, 40 cycles of denaturation at 95 °C for 10 s, annealing at 55–56 °C for 10 s, and extension at 72 °C for 10 s. The primers were designed and synthesized by Fuzhou Shangya Biotechnology Co., Ltd., Fuzhou, China. The primer sequences are shown in Table 2. GADPH was used as a reference gene, and the relative mRNA expression level was calculated using the 2^−△△CT^ method.

### 2.6. Bacterial DNA Extraction and 16S rRNA Gene Sequencing

Total bacterial DNA was extracted from each cecal content sample using the Stool DNA Kit (D4015-01, Omega Bio-tek, Norcross, GA, USA). The concentration and purity of DNA were determined by NanoDrop2000 (Thermo Scientific Corporation, Wilmington, NC, USA). The hypervariable region V3-V4 of the bacterial 16S rDNA was amplified with primer pairs 338F (5’-ACTCCTACGGGAGGCAGCAG-3’) and 806R (5’-GGACTACHVGGGTWTCTAAT-3’) by an ABI GeneAmp&reg 9700 PCR thermocycler (ABI, Los Angeles, CA, USA). Quantitative detection of PCR products was performed with QuantiFluor TM-ST Blue Fluorescence Quantitative System (Promega, Madison, WI, USA) according to the requirements for each sample sequencing amount. The library was constructed using the TruSeq^®^ DNA PCR-Free Sample Preparation Kit. The purified amplification products were sequenced on the NovaSeq-6000 platform (Illumina, San Diego, CA, USA). Amplicon sequence variants (ASVs) with 99% similarity cutoff delineated were clustered using divisive Amplicon Denoising Algorithm 2 (DADA2). ASV represents sequence and abundance information. The species taxonomy analysis, community diversity analysis, and species difference analysis were carried out, based on the representative sequence and abundance information of the ASVs.

### 2.7. Data Analysis and Statistics

Statistical analysis was performed using SPSS, version 25.0 (SPSS, Inc., Chicago, IL, USA). The Shapiro–Wilk test and Levene’s test were used to check the normality of the data and homogeneity of variance. The experimental data were analyzed using one-way analysis of variance (ANOVA) and Tukey’s multiple range tests for multiple comparisons. The results are presented as the mean ± standard deviation. *p* < 0.05 was considered statistically significant, and 0.05 ≤ *p* ≤ 0.10 was taken to indicate a statistical tendency.

## 3. Results

### 3.1. Growth Performance

The effects of NCCC supplementation on the growth performance of Ira white rabbits are presented in Supplementary Table S1 [37]. Compared with the CON group, ADG, and FBW were increased in the NCCC I, II, and III groups (*p* < 0.05). Supplementation with antibiotics or 200 mg/kg NCCC decreased the diarrhea rate compared with the CON group (*p* < 0.05). Dietary NCCC supplementation did not affect ADFI, IBW, FCR, or the death rate among the groups (*p* > 0.05).

### 3.2. The Content of Antioxidant Enzymes and the Expression of Related Genes in the Liver

As shown in Table 3, compared with the control group, the content of MDA in the livers of the rabbits was decreased in the NCCC I and NCCC II groups. Interestingly, the content of MDA in the NCCC III group was higher than that in the NCCC I and NCCC II groups. Similarly, the content of Cu/Zn-SOD was decreased in the NCCC I and NCCC II groups compared with the control group, and the content of Cu/Zn-SOD in the NCCC III group increased compared with the NCCC I and NCCC II groups. Compared with the control and the SAL group, dietary supplementation with 50 mg/kg and 100 mg/kg NCCC decreased the CER content. There were no differences in GSH-Px concentration among groups (*p* > 0.05). Furthermore, the gene expression of *CER* in the liver of the Ira rabbits in the SAL, NCCC I, and NCCC II groups was lower than that in the CON group (*p* < 0.05), and the gene expression of *Cu/Zn-SOD* was increased in the SAL and NCCC III groups compared with the CON, NCCC I, and NCCC II groups (*p* < 0.05) (Table 4).

### 3.3. The Content of Immune Factors in Jejunal Mucosa and the Expression of Related Genes

**Figure 1:**
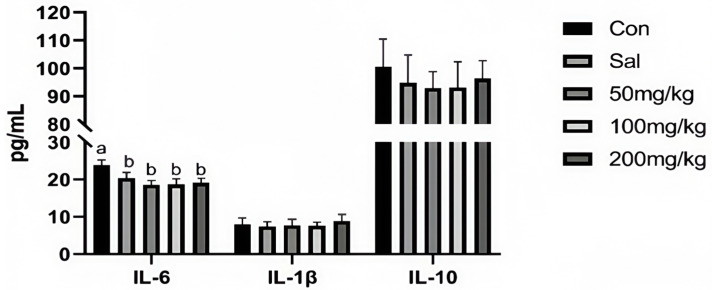
Effect of NCCC on the content of cytokines in jejunal mucosa of 28-day weaned Ira white rabbits. Con = control group with the basal diet; Sal = the basal diet +60 mg/kg salinomycin; 50 mg/kg = basal diet +50 mg/kg NCCC; 100 mg/kg = basal diet +100 mg/kg NCCC; 200 mg/kg = basal diet +200 mg/kg NCCC; IL-6 = Interleukin-6; IL-1β = Interleukin-1 beta; IL-10 = Interleukin-10. ^a, b^ Values with no letter or the same letter superscripts have no significant difference (*p* > 0.05), while with different lowercase letters indicate a significant difference (*p* < 0.05). Mean values are based on 6 replicates per treatment with 6 rabbits per replicate.

**Figure 2:**
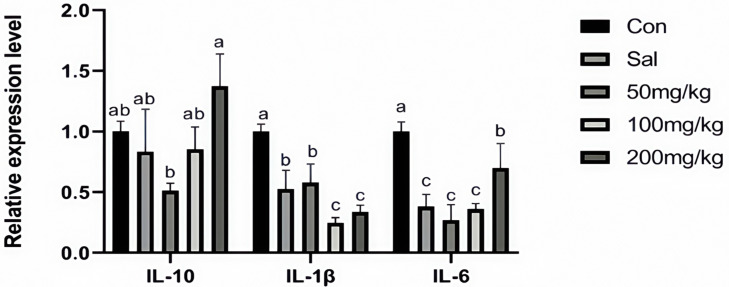
Effect of NCCC on gene expression of inflammatory factors in jejunal mucosa weaned Ira white rabbits. Con = control group with the basal diet; Sal = the basal diet +60 mg/kg salinomycin; 50 mg/kg = basal diet +50 mg/kg NCCC; 100 mg/kg = basal diet +100 mg/kg NCCC; 200 mg/kg = basal diet +200 mg/kg NCCC; IL-6 = Interleukin-6; IL-1β = Interleukin-1 beta; IL-10 = Interleukin-10. ^a, b, c^ Values with no letter or the same letter superscripts have no significant difference (*p* > 0.05), while different lowercase letters indicate a significant difference (*p* < 0.05). Mean values are based on 6 replicates per treatment with 6 rabbits per replicate.

As illustrated in Figure 1, dietary NCCC and SAL supplementation decreased the content of IL-6 in the jejunum mucosa of rabbits (*p* < 0.05). However, it did not affect other immune factor parameters (*p* > 0.05). Figure 2 shows that the gene expression of *IL-10* was down-regulated in the NCCC I group compared with the NCCC III group (*p* < 0.05). Dietary supplementation with NCCC and SAL down-regulated the *IL-1β* gene expression (*p* < 0.05), and compared with the SAL and NCCC I groups, the gene expression of *IL-1β* was down-regulated in the NCCC II and III groups (*p* < 0.05). Compared with the CON group, the gene expression of *IL-6* was down-regulated with supplementation of NCCC and SAL (*p* < 0.05), and the gene expression of *IL-6* was up-regulated in the NCCC III group compared with the SAL, NCCC I, and NCCC II groups (*p* < 0.05).

### 3.4. Cecal Microbiota

#### 3.4.1. Amplicon Sequence Variant (ASV) Cluster Analysis and Rarefaction Curve

**Figure 3:**
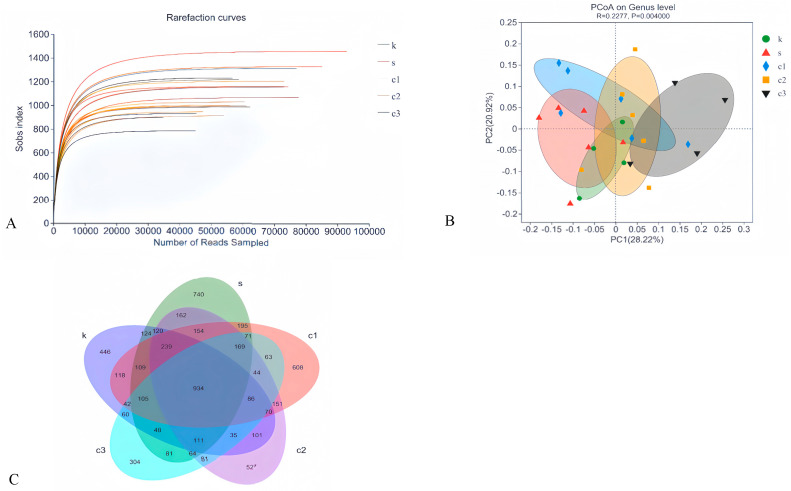
Modulation of cecal microbiota by five treatments. (**A**) Rarefaction curves of observed species. (**B**) PCoA analysis based on Bray–Curtis’s distance. (**C**) Venn diagram of ASVs. PCoA analysis based on Bray–Curtis’s distance. k = CON group with basal diet; s = SAL group with 60 mg/kg salinomycin; c1 = NCCC I group with 50 mg/kg NCCC; c2 = NCCC II group with 100 mg/kg NCCC; c3 = NCCC III group with 200 mg/kg NCCC.

To assess the effect of NCCC on the colonic microbiota of Ira rabbits, the colonic microbiota in the CON, SAL, NCCC I, NCCC II, and NCCC III groups were analyzed. A total of 3,577,886 valid data points with a quality control effectiveness rate of 94.01% were obtained from 16S rRNA sequencing of the colonic microbiota of Ira rabbits. The rarefaction curve reached a plateau after sequencing more than 40,000 reads per sample, indicating that the sequencing depth is sufficient to reflect the microbial diversity in the colon of Ira rabbits (Figure 3A). After clustering analysis of the total reads based on sequences with an identity of 97%, 2625 amplicon sequence variants were detected. The Venn diagram of cecal microbial ASVs (Figure 3C) showed that there were 934 shared core ASVs, and the number of unique ASVs was 466,740,608,527, with 304 in the CON, SAL, and NCCC I, II, and III groups, respectively.

#### 3.4.2. Beta-Diversity Analysis

Beta-diversity analysis, conducted using Principal Co-ordinates Analysis (PCoA), revealed no significant distinction in microbial community composition among the CON, SAL, and NCCC I and II groups (*p* > 0.05). However, a significant divergence was observed between the microbial communities of the CON and SAL groups compared with the NCCC III group (*p* < 0.05) (Figure 3B).

#### 3.4.3. Heat Map Analysis at the Phylum Level

**Figure 4:**
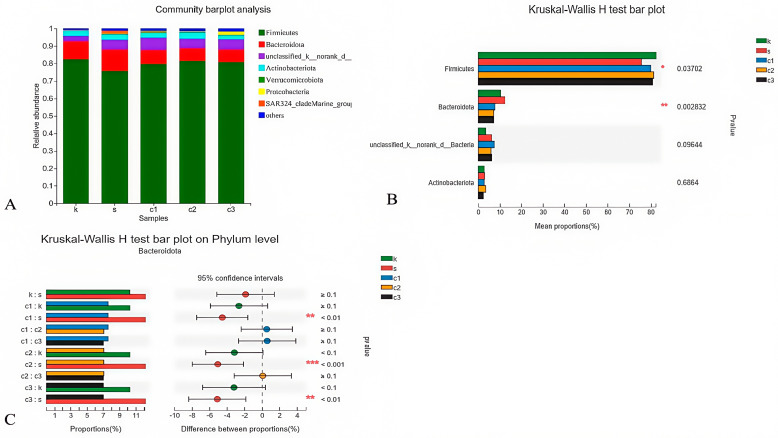
Modulation of cecal microbiota by five treatments. (**A**) Relative abundance at phylum level; (**B**) histogram, and (**C**) post hoc analysis of species phylum level difference test. k = CON group with basal diet; s = SAL group with 60 mg/kg salinomycin; c1 = NCCC I group with 50 mg/kg NCCC; c2 = NCCC II group with 100 mg/kg NCCC; c3 = NCCC III group with 200 mg/kg NCCC.

The composition of microbes in the cecum at the phylum level showed that Firmicutes and Bacteroidetes (92.54%) were identified as the predominant phyla in all experimental groups (Figure 4A). A significant difference was observed among the groups (*p* < 0.05), as depicted in Figure 4B. Furthermore, based on the Kruskal–Wallis H test, the dietary NCCC groups exhibited a significantly lower relative abundance of Bacteroidetes compared with the SAL group (*p* < 0.05), indicating a dietary influence on the cecal microbial composition (Figure 4C).

#### 3.4.4. Heat Map Analysis at the Genus Level

**Figure 5:**
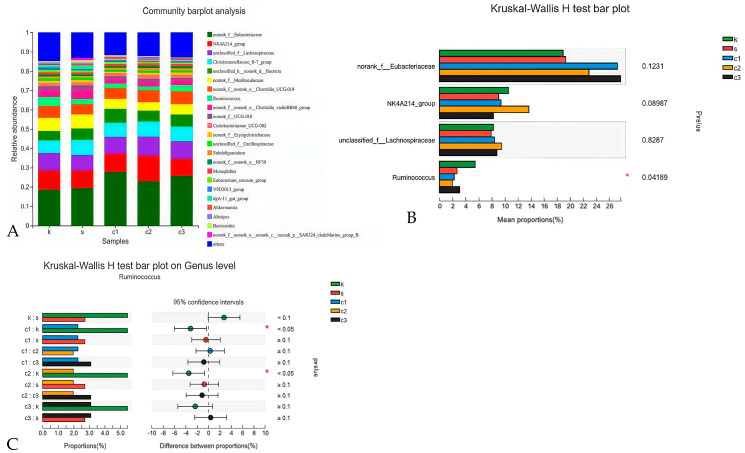
Modulation of cecal microbiota by five treatments. (**A**) Relative abundance at genus level in the cecal microbiota; (**B**) histogram and (**C**) post hoc analysis of species genus level difference test. k = CON group with basal diet; s = SAL group with 60 mg/kg salinomycin; c1 = NCCC I group with 50 mg/kg NCCC; c2 = NCCC II group with 100 mg/kg NCCC; c3 = NCCC III group with 200 mg/kg NCCC.

At the genus level, the unidentified *Eubacteriaceae*, unclassified *Lachnospiraceae*, *Christensenellaceae*, and *Ruminococcus* were the predominant genera of rabbits in each group (Figure 5A). Analysis of the relative abundance at the genus level revealed significant differences in the *Ruminococcus* among groups. (*p* < 0.05) (Figure 5B). To be specific, the relative abundance of *Ruminococcus* was lower in the NCCC I and NCCC II groups compared with the CON group based on the Kruska–Wallis H test (*p* < 0.05) (Figure 5C).

## 4. Discussion

The sudden changes in the feeding structure of weaned rabbits can easily cause oxidative stress, which increases the body’s susceptibility to disease, increases mortality, and ultimately leads to reduced economic benefits [38,39,40]. Copper (Cu), an essential micronutrient, exists in cells in either a reduced form (Cu^+^) or an oxidized form (Cu^2+^), which is crucial for both structural integrity and catalytic activity within biological systems [41]. Research has demonstrated that dietary copper can enhance the capacity of antioxidants in livestock and poultry [42,43]. In the present study, dietary supplementation with 50 and 100 mg/kg NCCC significantly decreased the content of liver MDA in rabbits. In addition, our findings showed an increase in the gene expression of Cu/Zn-SOD in the liver of Ira rabbits with the supplementation of 200 mg/kg NCCC. Malondialdehyde (MDA) is one of the final products of the lipid peroxidation process. MDA can damage cell membrane structure, destroy the function of the cell, and even induce apoptosis [44,45]. Therefore, MDA is a critical biomarker for assessing the extent of lipid peroxidation, oxidative stress, and cellular damage. Cu/Zn-SOD is an important antioxidant enzyme that catalyzes the conversion of superoxide radicals into molecular oxygen and hydrogen peroxide [46]. Copper is an essential cofactor of Cu/Zn-SOD for maintaining its activity [47]. The copper is transported into the cell by the CTR1 transporter and binds with the copper chaperone for superoxide dismutase (CCS), which delivers the copper to superoxide dismutase 1 (SOD1). It then plays a role in detoxifying reactive oxygen species (ROS) and maintaining copper homeostasis [48]. Consistent with our research findings, Mohammad et al. reported that dietary supplementation with 90 mg/kg nano-CuO improved the activity of SOD, GSH-Px, and T-AOC in the serum, and reduced the serum MDA content of broilers [49]. Similarly, El-kazaz et al. reported that the addition of 10 mg/L copper nanoparticles to drinking water increased the activity of SOD and decreased the content of MDA in the serum of broilers, enhancing their antioxidant capacity [50].

Interestingly, in the present study, dietary supplementation with 50 and 100 mg/kg NCCC decreased the levels of CER in the livers of the Ira rabbits. CER is a copper-containing α2-glycoprotein with antioxidant effects, which can inhibit ROS damage mediated by ferrous ions [51]. In addition, it is essential for copper transportation and iron metabolism [52]. Initially, the activity of antioxidant enzymes increases to balance the level of ROS, which leads to a reduction in endogenous antioxidants once the balance is achieved [53]. Therefore, we inferred that the oxidative stress was at a low-level state or had been eliminated in the body of 28-day weaned Ira white rabbits. However, when the body’s redox balance is disturbed, copper in the blood or tissue may again show protective properties against oxidative stress [54]. In addition, the excess copper can be combined with CER to enter the bile and is excreted from the body, reducing the toxicity of metal accumulation on the organism and avoiding tissue damage and dysfunction [55]. This mechanism may also account for the observed decrease in CER levels in the present study, thereby helping to avert the detrimental effects of excessive copper on the organism.

Copper is an essential trace element in the homeostasis of the immune system, playing a crucial role in the functioning of the immune response [56]. Copper deficiency profoundly inhibits immune cell development and impairs their functions, leading to alterations in the production and secretion of immune mediators [57,58]. A previous study showed that dietary copper increased the levels of neutrophils and monocytes in pigs reared in an aseptic environment [59]. Wang et al. reported that dietary supplementation with 100 mg/kg copper-loaded chitosan nanoparticles (CNP-Cu) increased the content of IgA, IgG and IgM, as well as complement C3 and C4, in the serum, enhancing the immune function of broilers [31]. In the present study, dietary supplementation with NCCC decreased the level of IL-6 in the jejunum of the Ira rabbits. Furthermore, dietary NCCC inhibited the gene expression of IL-1β. Interleukin 6 (IL-6), a member of the pro-inflammatory cytokine family, plays an essential role in modulating inflammation. Upon tissue damage, infection, or other stimuli, immune cells will generate IL-6, such as macrophages, monocytes, and non-immune cells including endothelial cells, mesenchymal cells, and fibroblasts [60,61]. Subsequently, IL-6 actively participates in a range of acute inflammatory diseases. However, the persistent production of IL-6 exerts detrimental effects on acute systemic inflammatory response syndrome and chronic immune-mediated disorders [62]. Similarly, the cytokine Interleukin-1β (IL-1β) is also a pro-inflammatory cytokine in the inflammatory response process, and its excessive release can trigger a cascade of inflammatory responses [63]. Therefore, the levels of IL-6 and IL-1β are higher during infection and inflammation. Consistent with our results, copper/zinc-loaded montmorillonite supplementation decreased MDA concentrations in the jejunum and ileum, and also decreased intestinal IL-1β, IL-6, and TNF-α levels, promoting the growth performance of weaned piglets [64]. However, Wu et al. reported that dietary copper supplementation increased serum IL-6 and enhanced the immune function of broilers [65], which is contrary to our results. IL-6 has also been reported to induce the production of anti-inflammatory cytokines such as IL-1 receptor antagonists and soluble TNF receptors, and in chronic inflammatory or autoimmune diseases, high levels of IL-6 may instead act to suppress inflammation [66,67]. Therefore, we hypothesized that the differences in test results might be related to the immune status of different feeding subjects, and the specific mechanism still needs to be further explored.

Gut microbiota play an important role in the physical health of animals. Under normal physiological conditions, the intestinal microflora is in a dynamic equilibrium state of interdependence and mutual constraints [68]. However, the digestive system of weaning rabbits is not fully developed, which may create susceptibility for pathogenic bacteria to infiltrate the intestinal tract, leading to a significant dysregulation of the intestinal microbiota [7,69]. Furthermore, the transition from breast milk to solid feeds impacts the intestinal microbiota, potentially leading to intestinal inflammation, and diarrhea [70,71]. Studies have shown that dietary copper modulates the intestinal microbiota structures [72,73]. In the present study, we found that dietary supplementation with 200 mg/kg NCCC modified the intestinal microbiota structures in the cecal of rabbits. The present study revealed that the phyla Firmicutes and Bacteroides were predominant in the gut microbiota of weaning rabbits, consistent with previous findings [74,75]. Additionally, dietary supplementation with copper has been demonstrated to modulate the intestinal microbiota by enhancing the proliferation of beneficial intestinal bacteria [76]. Forouzandeh et al. reported that dietary supplementation with 150 mg/kg copper oxide (CuO_2_) increased the abundance of beneficial digestive streptococcidae and *Clostridium* in the ileum of broilers, while concurrently reducing colonization by potentially pathogenic bacteria [77]. Similarly, Li et al. found that dietary copper/zinc montmorillonite enhanced the relative abundance of the core bacteria *Lactococcus* and *Bacillus* in the colon of weaned piglets and reduced *Streptococcus* and *Pseudomonas*, which are potentially pathogenic bacteria [78]. Nevertheless, Nguyen et al. reported that dietary copper supplementation non-selectively down-regulated the relative abundance of beneficial or pathogenic bacteria, including *Lactobacillus*, *Bacteroides*, and *Enterobacteriaceae*, in the cecal microbiota of broilers [79]. Additionally, Du et al. found that dietary supplementation with organic copper in the diet decreased the relative abundance of *Ruminococcus* in the cecum and significantly reduced the content of short-chain fatty acids in the cecal of rabbits [80]. In the present study, dietary NCCC reduced the abundance of *Bacteroides* and *Ruminococcus* in the cecal of Ira rabbits. Numerous studies have demonstrated that *Ruminococcus* degrades cellulose and hemicellulose and produces short-chain fatty acids (SCFAs), maintaining intestinal health [81]. However, an elevated concentration of SCFA may increase intestinal permeability, potentially leading to diarrhea [82]. SCFA has been reported to stimulate the release of 5-hydroxytryptamine (5-HT) from intestinal mucosal cells, thereby increasing intracellular Ca^2+^ concentration, activating K^+^ channels to induce hyperpolarization and accelerate the contraction of intestinal smooth muscle, which may contribute to the excretion of feces [83]. Du et al. reported that SCFAs were reduced in the intestines of rabbits in the organic Cu group, which may contribute to a decrease in fecal moisture, resulting in slower and drier excretion, potentially ameliorating the symptoms of diarrhea [80]. In our previous study, a comprehensive assessment of diarrhea based on a diarrhea rating scale recording the daily diarrhea status of rabbits and a combination of whether the animals exhibited symptoms such as rough fur, lethargy and weight loss, we found that the addition of a diet with 100 mg/kg NCCC reduced the incidence of diarrhea in weaned Ira rabbits [37]. However, the relationship between the observed outcome and the reported mechanism involving high levels of SCFA that induced diarrhea warrants further investigation. Furthermore, the different results of copper on the intestinal microbial flora might be caused by discrepant doses, inconsistency of form, model animal type, or trial period in these studies. Therefore, the mechanism of influence of dietary NCCC supplementation on the gut microbial flora needs to be further investigated. 

It is worth mentioning that other studies reported that plant-based extracts used as feed additives, including *Ganoderma lucidum* polysaccharide peptides, astragalus polysaccharides, etc., could also enhance antioxidant and immune functions and promote intestinal health through up-regulation of antioxidant enzymes, increase the expression level of tight junction proteins and regulatory immune factors, and regulate the diversity of cecal flora, which could lead to the enhancement of livestock and poultry production performance [84,85,86,87]. Consistently, our results indicate that NCCC also has similar functional effects to other replacement antioxidant product additives, and in comparison, NCCC or metal nanoparticle feed additives may be more suitable for situations that require intensive nutritional supplementation. Taken together, the application of NCCC in actual production still needs to be investigated or synergized with other natural alternative antibiotic products.

## 5. Conclusions

In conclusion, dietary supplementation with NCCC in the diet of Ira white rabbits could enhance antioxidant and immune functions and regulate the dynamic balance of intestinal flora, reducing the diarrhea rate and promoting the health of Ira white rabbits.

## Figures and Tables

**Table 1:** Composition and nutrient levels of basal diets (air-dry basis, %). The nutrient levels are based on the Meat Rabbits Feeding Standards (DB37/T 1835-2011) [36].

Items	Content
Ingredients	
Alfalfa meal	38.00
Corn	9.00
Wheat bran	16.90
Wheat DDGS	6.00
Rice husk powder	9.00
Soybean meal	5.00
Rice bran meal	10.00
Wheat middling	3.00
Limestone	1.20
Methionine	0.10
Lysine	0.20
NaCl	0.60
Premix ^1^	1.00
Total	100.00
Nutrient levels ^2^	
Digestible energy/(MJ/kg)	9.96
Crude protein	15.48
Crude fiber	17.35
Neutral detergent fiber	33.56
Acid detergent fiber	20.51
Acid detergent lignin	5.90
Calcium	0.90
Total phosphorus	0.51
Lysine	0.74
Methionine + Cystine	0.51

^1^ The premix provided the following (per kilogram of the diet): vitamin A, 12,000 IU; vitamin D3, 900 IU; vitamin E, 50 mg; vitamin K3, 1 mg; vitamin B1, 1 mg; vitamin B2, 3 mg; vitamin B6, 1 mg; vitamin B12, 0.01 mg; nicotinic acid, 30 mg; pantothenic acid, 8 mg; folic acid, 0.2 mg; biotin, 0.08 mg; choline chloride, 100 mg; copper, 10 mg; ferrous, 50 mg; manganese, 8 mg; zinc, 50 mg; iodide, 1 mg; selenium, 0.05 mg; cobalt, 0.25 mg. ^2^ Nutrient levels are calculated values.

**Table 2:** Nucleotide sequences of primers for quantitative real-time PCR assay.

Gene	Primer Sequence (5′→3′)	Gene Bank
*GADPH*	F:5′-GGTAGTGAAGGCTGCTGCTGATG-3′	NC_013676.1
	R:5′-GTCTCGCACTCCAATCTCTGTTCC-3′	
*SOD1*	F:5′-AGTTCGACACCGACCTGAAG-3′	NC_013682.1
	R:5′-TCCTGGTATTGAGGGCTGTC-3′	
*CER*	F:5′-TATGGCAACAGAGTGGCTCG-3′	NC_013682.1
	R:5′-GCTGGGTGGGTAGGATGTTT-3′	
*IL-1β*	F:5′-TCTGCAACACCTGGGATGAC-3′	NC_013670.1
	R:5′-TCAGCTCATACGTGCCAGAC-3′	
*IL-6*	F:5′-ACGATCCACTTCATCCTGCG-3′	NC_013678.1
	R:5′-GGATGGTGTGTTCTGACCGT-3′	
*IL-10*	F:5′-TCACCGATTTTCTCCCCTGTG-3′	NC_013684.1
	R:5′-ATGTCAAACTCATGGCTT-3′	

**Table 3:** Effects of NCCC on the content of antioxidant enzymes in the liver of Ira rabbits.

Items ^2^	Groups ^1^					*p*-Value
	CON	SAL	NCCC I	NCCC II	NCCC III	
MDA (nmol/mg)	1.979 ± 0.115 ^a^	1.850 ± 0.310 ^ab^	1.594 ± 0.162 ^b^	1.627 ± 0.232 ^b^	1.726 ± 0.173 ^ab^	0.000
CER/(U/L)	17.39 ± 1.41 ^a^	17.09 ± 2.56 ^a^	14.72 ± 0.77 ^b^	14.98 ± 1.41 ^b^	16.38 ± 1.24 ^ab^	0.004
GSH-Px (pg/mL)	29.37 ± 1.56	28.09 ± 3.41	27.23 ± 1.50	27.13 ± 3.07	27.37 ± 1.72	0.134
Cu/Zn-SOD (pg/mL)	41.44 ± 0.81 ^a^	40.32 ± 6.13 ^ab^	35.64 ± 3.83 ^b^	37.29 ± 4.26 ^b^	39.29 ± 4.17 ^ab^	0.006

MDA = malondialdehyde; CER = ceruloplasmin, GSH-Px = glutathione peroxidase; Cu/Zn-SOD = copper/zinc superoxide dismutase. ^1^ Control group (CON, basal diet); Antibiotic (salinomycin) group (SAL, basal diet +60 mg/kg salinomycin); NCCC I group (basal diet +50 mg/kg NCCC); NCCC II group (basal diet +100 mg/kg NCCC); NCCC III group (basal diet +200 mg/kg NCCC). ^2^ Values are the mean ± SD, *n* = 6. ^a, b^ Values with no letter or the same letter superscripts have no significant difference (*p* > 0.05), while different lowercase letters indicate a significant difference (*p* < 0.05).

**Table 4:** Effects of NCCC on the gene expression of antioxidant enzymes in the liver of Ira rabbits.

Items ^2^	Groups ^1^					*p*-Value
	CON	SAL	NCCC I	NCCC II	NCCC III	
CER/(U/L)	1.00 ± 0.25 ^a^	0.51 ± 0.13 ^b^	0.46 ± 0.11 ^b^	0.43 ± 0.19 ^b^	0.60 ± 0.17 ^ab^	0.015
Cu/Zn-SOD/(pg/mL)	1.00 ± 0.46 ^b^	1.99 ± 0.57 ^a^	1.10 ± 0.32 ^b^	1.81 ± 0.27 ^b^	1.98 ± 0.27 ^a^	0.025

CER = ceruloplasmin; Cu/Zn-SOD = copper/zinc superoxide dismutase. ^1^ control group (CON, basal diet); Antibiotic (salinomycin) group (SAL, basal diet +60 mg/kg salinomycin); NCCC I group (basal diet +50 mg/kg NCCC); NCCC II group (basal diet +100 mg/kg NCCC); NCCC III group (basal diet +200 mg/kg NCCC). ^2^ Values are the mean ± SD, n = 6. ^a, b^ Values with no letter or the same letter superscripts have no significant difference (*p* > 0.05), while different lowercase letters indicate a significant difference (*p* < 0.05).

## Data Availability

Most of the data generated or analyzed in this study are presented in this published article or its Supplementary Information. Additional data not included here are accessible upon reasonable request to the corresponding authors.

## Supplementary Materials

The following supporting information can be downloaded at: https://www.mdpi.com/article/10.3390/ani15020184/s1, Table S1: Effects of NCCC on the growth performance of weaned Ira rabbits. Supplementary material associated with this article can be found in the online version at https://link.cnki.net/urlid/11.5461.S.20230620.1555.038.
