# Supplementary material for: Effects of Dietary Nano-Composite of Copper and Carbon on Antioxidant Capacity, Immunity, and Cecal Microbiota of Weaned Ira White Rabbits
Source: Animals (Basel). 2025 Jan 11;15(2):184. doi: 10.3390/ani15020184 (PMC11758615; doi:10.3390/ani15020184)
Supplement: Supplementary file 1 [file animals-15-00184-s001.zip › animals-3373268-supplementary.pdf]

**Supplementary Materials:**

**Table S1 Effects of NCCC on the growth performance of weaned Ira rabbits**

| **Items2** | **Groups1** | | | | | **P-value** |
|---|---|---|---|---|---|---|
| | **CON** | **SAL** | **NCCC Ⅰ** | **NCCC Ⅱ** | **NCCC Ⅲ** | |
| ADFI (g/d) | 98.14±3.26 | 103.69±3.01 | 102.97±3.02 | 103.97±2.05 | 104.99±5.18 | 0.094 |
| ADG (g d) | 24.71±1.86$^{b}$ | 27.86±3.83$^{ab}$ | 27.44±5.11$^{ab}$ | 27.88±3.90$^{ab}$ | 28.37±2.63$^{a}$ | 0.039 |
| IBW (g) | 698.25±8.89 | 696．50±7.27 | 689.98±12.42 | 699.73±9.38 | 704．85±14.70 | 0.238 |
| FBW (g) | 1 390.13±43.25$^{b}$ | 1 478.76±52.77$^{a}$ | 1 465.00±54.49$^{a}$ | 1 479.22±85.63$^{a}$ | 1 489.82±36.07$^{a}$ | 0.037 |
| FCR | 3.99±0.31 | 3.76±0.39 | 3.86±0.76 | 3.80±0.59 | 3.72±0.33 | 0.892 |
| Death rate (%) | 29.17 | 14.58 | 18.75 | 12.5 | 8.33 | 0.069 |
| Diarrhea rate (%) | 4.81$^{a}$ | 2.32$^{b}$ | 3.15$^{ab}$ | 3.14$^{ab}$ | 2.54$^{b}$ | 0.002 |

ADFI = average daily feed intake; ADG = average daily gain; IBW = initial body weight; FBW = final body weight; FCR = feed conversion ratio.

1 Control group (CON, basal diet); Antibiotic group (SAL, basal diet + 60 mg/kg salinomycin); NCCC Ⅰ group (basal diet + 50 mg/kg NCCC); NCCC Ⅱ group (basal diet + 100 mg/kg NCCC); NCCC Ⅲ group (basal diet + 200 mg/kg NCCC).

2 Values are the mean ± SD, $n$ =6. $^{a, b}$ Values with no letter or the same letter superscripts have no significant difference ($p$ > 0.05), while different lowercase letters indicate a significant difference ($p$ < 0.05)
